# Functional Biomaterials for Treatment of Chronic Wound

**DOI:** 10.3389/fbioe.2020.00516

**Published:** 2020-06-03

**Authors:** Xi Zhang, Wentao Shu, Qinghua Yu, Wenrui Qu, Yinan Wang, Rui Li

**Affiliations:** ^1^Department of Hand Surgery, The Second Hospital of Jilin University, Changchun, China; ^2^Department of Burn Surgery, The First Hospital of Jilin University, Changchun, China; ^3^Department of Biobank, Division of Clinical Research, The First Hospital of Jilin University, Changchun, China; ^4^Key Laboratory of Organ Regeneration and Transplantation, The First Hospital of Jilin University, Changchun, China

**Keywords:** chronic wound, functional biomaterials, immunoregulation, antioxidant, angiogenesis

## Abstract

The increasing number of patients with chronic wounds caused by diseases, such as diabetes, malignant tumors, infections, and vasculopathy, has caused severe economic and social burdens. The main clinical treatments for chronic wounds include the systemic use of antibiotics, changing dressings frequently, operative debridement, and flap repair. These routine therapeutic strategies are characterized by a long course of treatment, substantial trauma, and high costs, and fail to produce satisfactory results. Biomaterial dressings targeting the different stages of the pathophysiology of chronic wounds have become an active research topic in recent years. In this review, after providing an overview of the epidemiology of chronic wounds, and the pathophysiological characteristics of chronic wounds, we highlight the functional biomaterials that can enhance chronic wound healing through debridement, anti-infection and antioxidant effects, immunoregulation, angiogenesis, and extracellular matrix remodeling. It is hoped that functional biomaterials will resolve the treatment dilemma for chronic wounds and improve patient quality of life.

## Introduction

Owing to an increasing number of patients with chronic diseases such as diabetes, there are more than 6.5 million patients with chronic wounds worldwide, and the overall medical cost for treating chronic wounds exceeds 25 billion US dollars per year (Sen et al., 2009). Chronic diseases including diabetes, cardiovascular and cerebrovascular diseases, hypoxia, cancer, and immunosuppression are causes of chronic wounds, as are local vascular disease, infection, and repetitive trauma (Morton and Phillips, 2016). According to the etiology, the categories with the highest incidence of chronic wounds are diabetic foot ulcers (DFUs), venous leg ulcers, and pressure ulcers. DFUs alone occur in more than 450 million adult diabetes patients, and there is a 25% lifetime chance of developing diabetes (Boulton et al., 2005; Cho et al., 2018). The high prevalence, high amputation rate, and high recurrence rate of DFUs cause a serious economic and social burden (Boulton et al., 2005; Cho et al., 2018). However, innovations in clinical treatment methods and wound care have been insufficient for the past century (Harding, 2015). Familiarity with the anatomical structure and function of the skin, as well as the difference between acute wound healing and chronic wound healing, is essential for the treatment of chronic wounds.

The anatomy of normal skin is mainly composed of epidermis, dermis, and subcutaneous tissue. The avascular epidermis layer is closely connected to the dermis layer through the basement membrane, and the barrier function and prevention of fluid loss of the skin are mainly provided by the stratum corneum structure in the epidermal. Dermal matrix components, predominantly collagen (Col) and elastin, are derived from fibroblasts. The main cell types of the subcutaneous tissue are fibroblasts and adipocytes (Mathes et al., 2014).

Epidermal stem cells located in the epidermis, sweat glands, and sebaceous glands can effectively promote skin regeneration under physiological and pathological conditions (Snippert et al., 2010). To ensure the continuity of the skin structure and the integrity of its function, a dynamic and highly controlled recovery process can be initiated after injury. This healing process, which involves different types of cells and intracellular and intercellular signaling pathways, has been extensively studied (Gurtner et al., 2008). The healing process of acute wounds in healthy individuals is rapid and orderly. This process consists of four sequential and partially intertwined stages, hemostasis, inflammation, proliferation, and tissue remodeling, which represent the repair principles of most tissues (Gurtner et al., 2008; Gonzalez et al., 2016). The fibrin network formed during the hemostatic phase not only helps to enhance the barrier function but also promotes cell migration and fibroblast proliferation. Increased local penetration of neutrophils, lymphocytes, and monocytes represents the entry into the inflammatory phase, which generally lasts less than a week. During the proliferation phage, after the keratinocytes migrate to the damaged dermis, granulation tissue replaces the fibrin network formed during the hemostasis period. This new matrix is conducive to the maturation of keratinocytes, which completes the epithelialization process. The tissue remodeling period can start 2 weeks after injury, and the duration may be up to 1 year. The cells activated in the previous stage are reduced, and the conversion of type III Col to type I Col under the action of matrix metalloproteinases (MMPs) can enhance the repair of skin tissue during this stage (Gurtner et al., 2008; Gonzalez et al., 2016). The outcomes of acute wound healing can be classified as regeneration or repair, that is, epidermal-specific tissue replacement or repair with scar tissue, respectively (Reinke and Sorg, 2012).

If the healing process does not proceed smoothly, the wound will evolve into a chronic wound (Nunan et al., 2014). It is generally believed that chronic wounds cannot be repaired in a normal, orderly, and timely manner. In humans, anatomical and functional integrity cannot be restored within 3 months (Nunan et al., 2014). The physical space-occupying effect of the increase in local necrotic tissue, infection caused by various pathogens, poor local vascular conditions, and excessive levels of pro-inflammatory cytokines, proteases, and reactive oxygen species (ROS), etc. can all cause a wound to stagnate at the inflammatory reaction stage, resulting in delayed healing, or failure to heal. This may occur even after some tissue remodeling in partial wounds, which often leads to severe scar hyperplasia or keloids (Nunan et al., 2014; Zhao et al., 2016). There are clinical treatments for these complications, including systemic application of antibiotics, frequent changing of dressings, operative debridement, and flap repair. Other therapeutic techniques include the application of hyperbaric oxygen therapy (HBOT) to improve the wound microenvironment by increasing local oxygen content, the use of negative pressure-assisted wound therapy (RNPT) to improve the local blood supply, and the use of platelet-rich plasma (PRP) that is rich in cytokines and nutritional factors to treat chronic wounds (O'reilly et al., 2011; Topaz, 2012; Powers et al., 2016; Weller et al., 2019). However, the conventional treatment methods have the several disadvantages, including the need for anesthesia in patients undergoing operative debridement, the side effects of systemic application of antibiotics, and the repair of chronic wounds caused by flaps and skin grafts. HBOT, RNPT, and PRP only serve as adjuvant treatments for wounds. In short, all the above treatment methods fail to change the microenvironment of chronic wounds, which is not conducive to repair, and thus they do not represent breakthroughs in the treatment of chronic wounds. In addition, progress in wound care over the past century has been very slow (Harding, 2015).

**Scheme 1:**
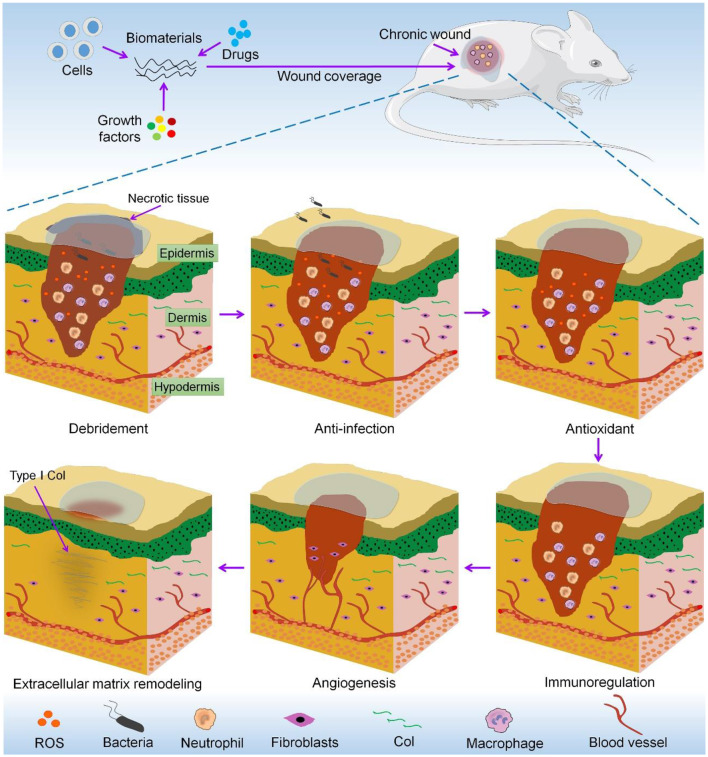
Functional biomaterials for repair of chronic wounds.

In view of the limitations of traditional treatments, biomaterials have received extensive attention regarding their potential applications in chronic wounds. Many commercial wound dressings, such as alginate and hydrocolloid-based dressings, shorten the healing time of chronic wounds by maintaining a moist microenvironment that can maintain cell activity and accelerate debridement and vascularization, and have been shown to have therapeutic potential (Field and Kerstein, 1994; Serena et al., 2016; Jones et al., 2018). Protease-modulating matrix products have also shown promise in the treatment of chronic wounds (Jones et al., 2018). In view of the increasing number of basic research studies of the treatment of chronic wounds with biomaterials, it is necessary to systematically summarize the functionalization of biomaterials. In this review, we focus on research progress with respect to biomaterials used for debridement and to achieve anti-infection and antioxidation effects, immunoregulation, angiogenesis, and extracellular matrix (ECM) remodeling in chronic wounds. A schematic of this review is shown in Scheme 1.

## Functional Biomaterials for Treatment of Chronic Wounds

The main role of functional biomaterials in the treatment of chronic wounds is to promote skin repair by adjusting the microenvironment of the wound (Kruse et al., 2015). Here, we introduce the characteristics of various functional biomaterials, their mechanisms, and their effects on chronic wound healing. Modern functional biomaterial dressings for chronic wounds are summarized in Table 1.

**Table 1:** Modern functional biomaterial dressings for chronic wounds.

**Function**	**Material**	**Bioactive agent**	**Model/Disease**	**Results**	**References**
Debridement	Sterigel®		Patients with pressure ulcers	The debridement effect was similar to that of a traditional carboxymethyl cellulose-based hydrogel, but the ability to absorb exudates was limited	Williams, 1997
	Polyacrylate	Protease	Patients with venous ulcers	Compared with an amorphous hydrogel, protease/polyacrylate more effectively removed necrotic tissue and granulation tissue.	Humbert et al., 2014
Anti-infection	CuS nanodots		0.7 cm circular wounds in diabetic mice	Non-healing MRSA-infected wounds treated with CuS nanodots combined with laser irradiation were healing after 12 days	Qiao et al., 2019
	AgNP/PAAS CMC	Calculus bovis	1.0 cm circular wounds in diabetic rats	The two-stage dressing promoted tissue repair in S. aureus-infected wounds	He et al., 2017
	TPP-loaded Tecophilic®		Patients with chronic leg ulcers	Reduced the area of chronic ulcers and effectively reduced pain	Arenbergerova et al., 2012
Antioxidant	PPCN	SDF-1	Splinted 0.6 cm circular wounds in diabetic mice	The antioxidant PPCN hydrogel could promote epidermal and appendage regeneration, combined with SDF-1 to further improve the repair effect; wounds were closed by day 24	Zhu et al., 2016
	Alginate	Edaravone	0.5 cm circular wounds in diabetic rats	The therapeutic effect was dose-dependent, which proved the dual role of ROS in chronic wound healing.	Fan et al., 2019
	PEI_25K_, Ceria, Col	AntagomiR-26a	1.5 cm circular wounds in diabetic rats	The PCN-miR/Col treatment promoted wound healing in diabetic rats, and the quality of repaired skin including Col and skin appendage was similar to that of normal skin	Wu et al., 2019
Immunoregulation	KSiNPs		0.7 cm circular wounds in diabetic mice	KSiNPs group promoted wound healing in diabetic mice through M2 macrophage polarization	Gan et al., 2019
	PVA/CS	Bee venom	1.8 cm circular wounds in diabetic rats	Wounds in the bee venom loaded PVA/CS hydrogel group were basically healed in day 21, and animals had lower IL-6 levels	Amin and Abdel-Raheem, 2014
Angiogenesis	HA	VEGF- plasmid	Splinted 0.6 cm circular wounds in diabetic mice	HA hydrogel with pores of 60 μm in diameter had the strongest granulation formation and healing ability, but the combination of VEGF plasmids did not further enhance the regeneration of granulation tissue	Tokatlian et al., 2015
	GH	IL-8, MIP-3α	1.0 cm circular wounds in diabetic mice	IL-8-loaded GH hydrogel exhibited stronger repair-promoting neovascularization, and wound healing ability than the MIP-3α treatment	Yoon et al., 2016
	PLLA, silica NPs	DMOG	0.8 cm circular wounds in diabetic mice	The treatment effectively promoted wound healing and neovascularization in diabetic mice	Ren et al., 2018
ECM remodeling	Col		Patients with venous leg ulcers	Commercial ovine-derived Col dressing cured 50% of venous ulcers in 12 weeks	Liden and May, 2013
	Poly 2/DS, CS	siRNA	0.6 cm circular wounds in diabetic mice	In the MMP-9 gene silencing group, the content of type I Col increased and wound healing was accelerated	Castleberry et al., 2016

### Debridement

Debridement is one of the key processes in preparing chronic wound beds. Bacteria and toxins often accumulate in necrotic tissue, which is composed mainly of inactivated Col. The long-term presence of necrotic tissue can cause local or even systemic infections. Tight combination of the necrotic tissue and the wound bed also has a physical occupying effect, which seriously hinders wound healing. Clearing the necrotic tissue can reduce the burden of bacteria and abnormal-phenotype cells, reduce local edema, and normalize the microenvironment of the wound surface (Falanga, 2004). Despite the obvious advantages of wound debridement, the impact of multiple operative debridement procedures and the requirement for anesthesia have severely limited its wide application in patients with underlying diseases.

Hydrogels with different gel-forming mechanisms and components are among the most widely used biomaterials (Wang Y. et al., 2019; Zhang et al., 2019c; Lin et al., 2020), and also play an important part in chronic debridement. Hydrogel dressings achieve debridement by rehydration, molting, and removal of inactivated and necrotic tissue. Sterigel®, an amorphous hydrogel derived from corn bran, was used for debridement of wound necrotic tissue and sloughing as early as 1997 (Williams, 1997). Hydrogel dressings are not suitable for chronic wounds with substantial or continuous exudation, and their applications are limited by the water absorption capacity of the hydrogel. As carboxymethyl cellulose adsorption particles have strong water absorption capacity, hydrocolloid occlusive dressings composed of carboxymethyl cellulose and polyurethane are suitable for wounds with more exudate. When the hydrocolloid dressing comes into contact with the wound exudate, the hydrogel matrix forms a hydrogel layer after swelling with water; the moist milieu created by the hydrocolloid promotes debridement and stimulates the formation of granulation tissue (Eaglstein, 2001). One meta-analysis of 12 randomized controlled trials including patients with venous ulcers and pressure ulcers showed that the healing rate of ulcers in the hydrocolloid dressing group was higher than that in the traditional gauze dressing group (Singh et al., 2004). Protease loaded polyacrylate-based dressing can also achieve effective debridement of chronic wounds (Humbert et al., 2014).

The effect of autolytic debridement after injury is often limited and the time is longer, the application of dressings with various debridement functions has also achieved good therapeutic effects in chronic wounds. Such dressings are especially suitable for patients in poor physical condition.

### Anti-infection

Chronic wounds have an increased risk of endogenous and exogenous infections. Infections lead to increased local or systemic inflammation, which is often an important cause of chronic wounds not healing or even deepening (Gjdsbl et al., 2006; Agostinho et al., 2011). Chronic wounds are often colonized by more than two bacterial species, the most common of which are *Staphylococcus aureus* and *Pseudomonas aeruginosa* (Gjdsbl et al., 2006). Bacteria in chronic wounds do not exist in a suspended state but colonize the ECM in the form of complex bacterial biofilms. This is often the reason for the emergence of drug-resistant bacteria, such as extended-spectrum β-lactamase-positive *Escherichia coli* (ESBL *E. coli*) and methicillin-resistant *S. aureus* (MRSA) (Suryaletha et al., 2018).

The treatment of chronic wounds with bacterial colonization generally involves interfering with the formation of new bacterial biofilms, removing the biofilms that have formed, and minimizing the use of antibiotics. A variety of natural and synthetic polymer-based antibacterial functional dressings, including types of hydrogel, foam, and fiber, have been shown to have anti-infection effects. Hydrogels are polymer solid materials with a three-dimensional (3D) network structure containing water as a dispersion medium. Chitosan (CS)-based dressings have many advantages, including excellent degradability and compatibility; they are also non-toxic and exhibits excellent antibacterial activity (Wang et al., 2012). In short, antibacterial CS dressings can effectively improve the local microenvironment of a wound (Archana et al., 2013); Ding et al. synthesized a series of polyurethane foam dressings with different cationic group contents, which showed antibacterial properties against both Gram-positive cocci and Gram-negative bacilli (Ding Y. et al., 2019); the uniquely high surface area to volume ratio and high porosity of fiber and electrospun nanofiber scaffolds enable them to effectively simulate the microenvironment of the ECM. Thus, such scaffolds are widely used in tissue repair, including in antibacterial treatments for chronic wounds (Ranjbar-Mohammadi et al., 2016; Ding J. et al., 2019; Feng et al., 2019a; Zhang et al., 2019a,b). Arenbergerova et al. prepared a polyurethane electrospun nanofiber dressing loaded with a photosensitizer, tetraphenylporphyrin (TPP). The activation of TPP by visible light produced cytotoxic singlet oxygen, which enhanced the antibacterial function of the dressing. The thin and transparent nature of this dressing maximized the photosensitizer activity (Arenbergerova et al., 2012).

**Figure 1:**
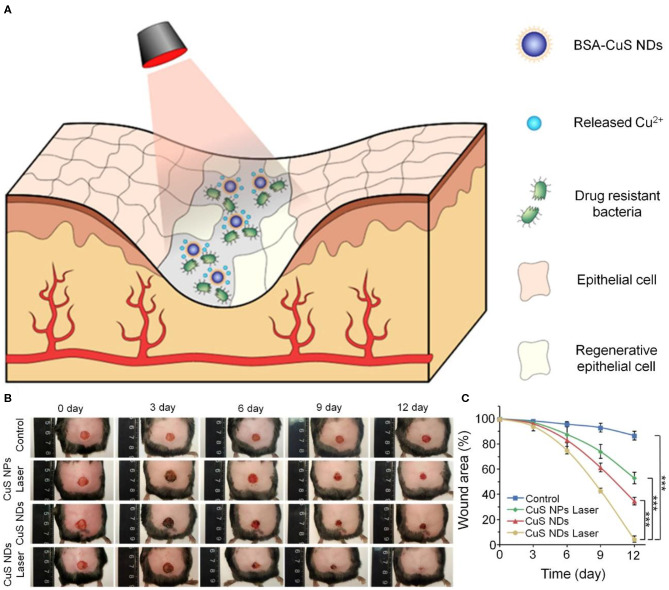
Antibacterial effect of laser-activated CuS nanodots. **(A)** Schematic diagram of CuS nanodots combined with laser irradiation to enhance antibacterial effect. **(B)** Wound images of MRSA-infected diabetic wound in mice. **(C)** Quantitative analysis of wound areas. ***p < 0.001. Reproduced with permission from Qiao et al. (2019).

Silver ion products such as silver sulfadiazine are excellent antibacterial agents and have been widely used in the treatment of burn wounds. However, they have shortcomings, including causing increased MMP expression. In recent years, silver nanoparticles (AgNPs) have been widely used in the treatment of chronic wounds owing to their broad-spectrum antimicrobial properties and safety. AgNPs inactivate pathogenic microorganisms by breaking disulfide bonds in proteins, thereby changing the tertiary structure of the proteins (Liau et al., 1997; Lara et al., 2011). Masood et al. fabricated AgNPs incorporated in a CS/poly(ethylene glycol) (PEG) hydrogel. Silver nitrate was reduced by CS and PEG solution to prepare the AgNPs, and then cross-linked with glutaraldehyde to produce the AgNP-loaded CS/PEG hydrogel. The incorporation of AgNPs increased the porosity and swelling capacity of the CS/PEG hydrogel. Further *in vivo* experiments confirmed that the AgNP-CS/PEG hydrogel effectively promoted the healing of diabetic wounds in rabbits (Masood et al., 2019). As one of the important trace elements, copper ions have attracted attention for their antibacterial function in chronic wounds. Qiao et al. developed protein-stabilized nano-copper sulfate (CuS) nanodots, using the photothermal effect of CuS to enhance their bactericidal properties. As shown in Figure 1, the synergistic action of the photothermal effect of the CuS nanodots and the release of copper ions produced a robust bactericidal effect on ESBL *E. coli* and MRSA *in vitro*. Further *in vivo* experiments used an MRSA-infected circular wound with a diameter of 0.7 cm in diabetic mice, and found that the experimental group basically healed after 12 days (Qiao et al., 2019).

Optimization of drug delivery systems is also an effective way to improve antibacterial performance. A cyclodextrin-based hydrogel was used as a drug delivery system to improve the efficacy of gallic acid, a polyphenol and broad-spectrum antimicrobial agent. The hydrophobic porosity and hydrophilic surfaces of cyclodextrin-based hydrogels improve their ability to encapsulate multiple molecules, which in this case helped to maintain the activity of gallic acid and enhance its antibacterial ability (Pinho et al., 2014). López-Iglesias et al. used CS/aerogel particles as carriers to enable sustained release of vancomycin, and achieved effective inhibition of MRSA while reducing the amount of antibiotic used and avoiding bacterial resistance (López-Iglesias et al., 2019). He et al. prepared a two-stage wound dressing, using nano silver and Calculus bovis as anti-infection and anti-inflammatory agents, respectively. Drug release experiments showed that the antibacterial phase occurred earlier than the long-duration drug release phase. Thus, the drug-loading system achieved rapid anti-infection, continuous reduction of inflammatory response, and accelerated healing of *S. aureus*-infected wounds (He et al., 2017).

### Antioxidant

The increase of the right amount of oxidant accelerates the healing of wounds in mice, as low concentrations of ROS, including superoxide anions ($\cdot {\text{O}}_{2}^{-}$), hydrogen peroxide (H_2_O_2_), and hydroxyl radicals (·OH), serve as signaling messengers to regulate gene expression. Low level of ROS are beneficial for maintaining cellular environmental homeostasis (Sen et al., 2002), whereas excessive ROS causes significant damage to nucleic acids and growth factors, and impairs angiogenesis in chronic wounds, which in turn hinders the healing process.

It has been proven that biomaterials and bioactive agents with antioxidative function, including diol-citrate esters, edaravone, and cerium dioxide, can accelerate chronic wound healing (Luo et al., 2004; Zhu et al., 2016; Speidel et al., 2017; Fan et al., 2019; Wu et al., 2019).

Zhu et al. fabricated a poly(polyethylene glycol citrate-*co*-*N*-isopropylacrylamide) (PPCN) hydrogel through continuous polycondensation and free radical polymerization reactions. The antioxidant diol-citrate esters endowed this temperature-sensitive PPCN hydrogel with antioxidant properties. Compared with the PBS group, the antioxidant PPCN hydrogel group more effectively promoted the regeneration of the epidermis and the appendages. Moreover, the PPCN content of the hydrogel could be adjusted to obtain better encapsulation and slow release of angiogenic chemokine stromal cell derived factor-1 (SDF-1), which achieved the desired functions of antioxidation and promotion of neovascularization (Zhu et al., 2016). Kim et al. reported that in the absence of biologically active agents, phytochemically stabilized gold nanoparticles increased the content of superoxide dismutase in acute wounds, with an antioxidant effect. They also showed that increasing angiopoietin 1 (ang1) and ang2 expression promoted angiogenesis in *in vivo* experiments (Kim et al., 2015). However, the effectiveness of these approaches in chronic wounds needs to be verified.

**Figure 2:**
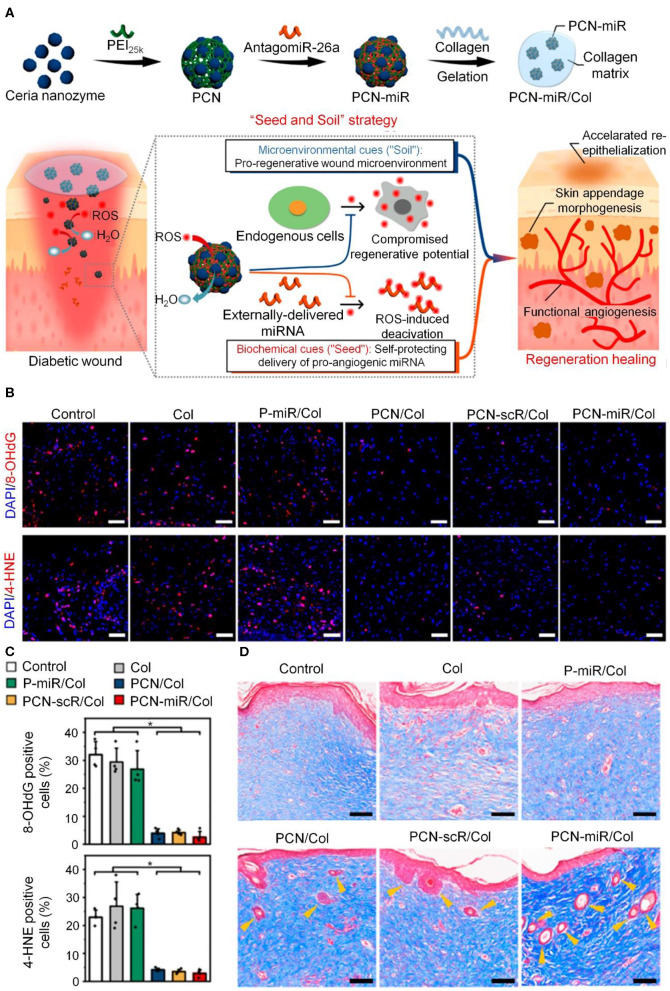
PCN-miR/Col hydrogel exerts antioxidant and neovascularization properties to promote healing of oxidative diabetes wound. **(A)** Schematic of PCN-miR/Col hydrogel preparation, antioxidation, and vascularization in chronic wounds. **(B)** *In vivo* results of ROS by immunofluorescence. **(C)** Quantitative analysis of results shown in **(B)**. *p < 0.05. **(D)** Masson staining images from day 28 post-operation. Scale bars, 100 μm. Reproduced with permission from Wu et al. (2019).

Edaravone, a commercial drug with the ability to scavenge ·OH, is used to treat cerebral ischemia and has also been used in chronic wounds. The therapeutic effect was dose-dependent, which proved the dual role of ROS in chronic wound healing (Fan et al., 2019). Ceria have also attracted attention as potential treatments for excessive-ROS-related diseases owing to their ability to mimic the activity of multiple antioxidant enzymes. miR-26a is an important microRNA that inhibits neovascularization in DFUs, only on the basis of removing excessive ROS, antagomiR-26a can effectively reverse the neovascularization inhibition caused by miR-26a and promote vascular regeneration. Wu et al. combined the use of antagomiR-26a and ceria nanozymes to treat non-healing wounds in diabetes patients. Ceria nanozymes with a diameter of 3 nm were prepared and covalently modified with 25 kDa polyethyleneimine (PEI_25K_) to produce PCN. PCN-miR was fabricated by electrostatic adsorption with negatively charged antagomiR-26a. The design of this drug delivery system ensured that antagomiR-26a had direct contact with the ceria nanozymes, preventing ROS from destroying its activity, as shown in Figure 2. *In vivo* experiments showed that this self-protecting hydrogel effectively promoted the repair of non-healing wounds in diabetes (Wu et al., 2019).

### Immunoregulation

The immune response is essential to clear pathogens in the early stages of wound healing. Toll-like receptors recognize signals from necrotic tissue and damaged ECM to activate tissue-resident macrophages and recruit other inflammation-related cells, including neutrophils and lymphocytes. Neutrophils produce antibacterial substances and secrete cytokines that promote angiogenesis to promote the recovery of tissues. However, a persistent inflammatory response can lead to the formation of chronic wounds (Percival et al., 2014). Furthermore, it is closely related to the activation type of macrophages in the wound microenvironment. Pro-inflammatory M1 macrophages aggravate tissue damage, whereas anti-inflammatory M2 macrophages are conducive to wound healing (Krzyszczyk et al., 2018).

**Figure 3:**
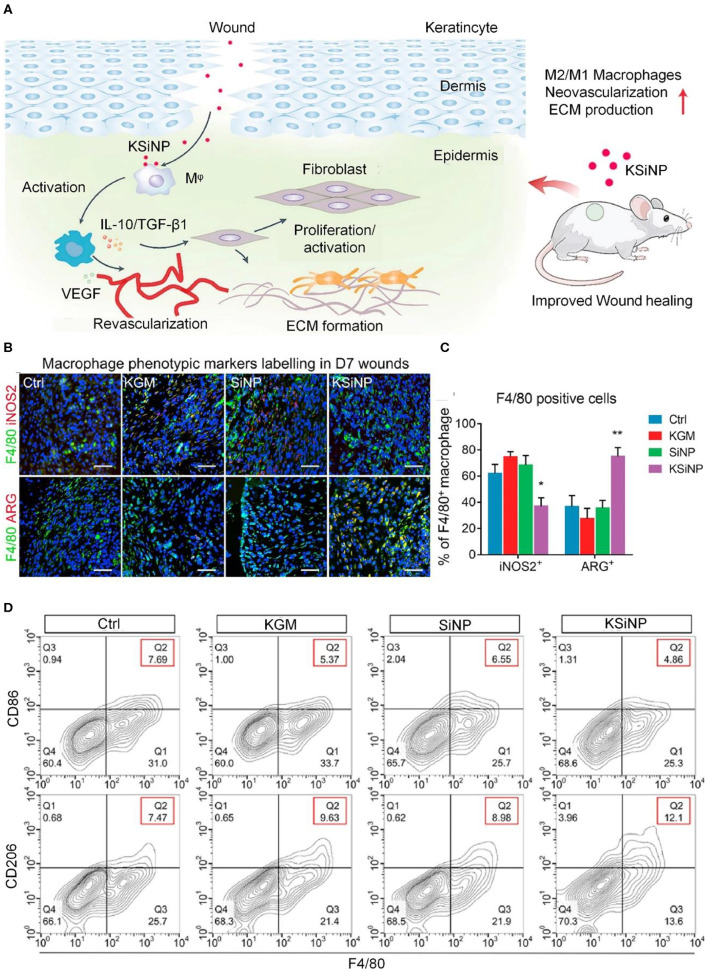
KSiNPs activate macrophages to differentiate into M2 phenotypes and promote wound healing. **(A)** Schematic of the mechanism by which KSiNPs promote chronic wound healing. **(B)** Immunofluorescence identification of macrophages in each experimental group 7 days post-operation. Scale bars, 20 μm. **(C)** Quantitative analysis of the results from **(B)**, showing that the proportion of M2 macrophages in the KSiNP group increased. *P < 0.05, **P < 0.01. **(D)** Flow cytometry detection of macrophage activation status in different groups of wounds on day 7. Reproduced with permission from Gan et al. (2019).

The polarization of M1 macrophages into M2 macrophages is an effective treatment method that can effectively reduce an excessive inflammatory response (Hesketh et al., 2017; Feng et al., 2019b). Therefore, the development of anti-inflammatory biomaterials based on immunoregulation is a research hotspot. As shown in Figure 3, studies have shown that konjac glucomannan-modified SiO_2_ nanoparticles (KSiNPs) can induce macrophages to differentiate into M2 phenotypes by inducing the accumulation of nano-clusters of the mannose receptor on the macrophage surface. Without containing any drugs, the material itself has an anti-inflammatory effect and promotes wound healing (Gan et al., 2019).

Proteases and excessive ROS produced by the immune response also hinder wound healing by inactivating growth factors (Sies, 1991; Schafer and Werner, 2008; Westby et al., 2018). Inhibiting the inflammatory response and reducing the cytokines produced during the inflammatory response are of great significance for improving the microenvironment of chronic wounds, and many biomaterial dressings or medicines with anti-inflammatory effects have been used in chronic wounds.

The anti-inflammatory effects of new hydrocellular foam dressings have been verified in acute wound models; they reduced the gene expression levels of inflammation-related factors including IL-1β, IL-6, and IL-10 (Banks et al., 1997; Yamane et al., 2015). Amin *et al*. compared the anti-inflammatory effect of Chinese medicine bee venom on chronic wounds with that of diclofenac hydrogel. They incorporated bee venom into a poly(vinyl alcohol) (PVA)/CS-based hydrogel using a freezing-thawing method. *In vivo* experiments showed that this bee venom-loaded PVA/CS hydrogel increased the gene expression levels of hydroxyproline and glutathione and reduced the level of IL-6, and its anti-inflammatory effect was comparable to that of the diclofenac hydrogel (Amin and Abdel-Raheem, 2014).

### Angiogenesis

Various conditions, such as hyperglycemia, cause endothelial dysfunction, resulting in insufficient blood supply to a chronic wound and an insufficient supply of nutrients and oxygen around the wound (Sorg et al., 2018). Therefore, reconstruction of the vascular network is essential to accelerate the healing of chronic wounds with local blood supply shortage. Impaired angiogenesis is associated with abnormal migration, proliferation, and regulation of endothelial progenitor cells. Some studies have confirmed that endogenous gas signaling molecules such as nitric oxide and hydrogen sulfide, which promote angiogenesis and granulation tissue formation, are closely related to wound healing (Durante, 2016; Malone-Povolny et al., 2019). In addition, insufficient blood supply will also affect the local skin temperature and thus the wound microenvironment. Acute wounds will increase the temperature of the wound under the mediation of certain factors, thereby causing vasodilation and achieving the purpose of providing more nutrition. Chronic wound blood flow is blocked in vascular disease, which reduces skin temperature and affects wound healing (Wilmore et al., 1977; Fierheller and Sibbald, 2010). At present, many kinds of dressings made using hydrogels, electrospinning, etc. with angiogenic effects have been used alone or in combination with angiogenic bioactive substances for the neovascularization of chronic wounds.

Hyaluronic acid (HA), a non-sulfated glycosaminoglycan, is the main component of ECM. HA promotes angiogenesis through controllable mild inflammation caused by degradation products (Toole and Slomiany, 2008; Gaffney et al., 2010; Hemshekhar et al., 2016). Tokatlian et al. used HA hydrogels with different pore sizes to carry a plasmid containing VEGF plasmids for the treatment of chronic wounds. When applied to a splinted wound in a diabetic mouse model, the hydrogel with pores of 60 μm in diameter had the strongest healing ability, but the combination of VEGF plasmids did not further enhance the regeneration of granulation tissue (Tokatlian et al., 2015). Gelatin, also known as hydrolyzed Col, has also been used as a drug delivery vehicle for chronic wound neovascularization. It has excellent biocompatibility and biodegradability, and low antigenicity (Yeh et al., 2011). Mesenchymal stem cells (MSCs) exert a chemotactic effect through paracrine signaling to promote the regeneration of chronic wounds. In order to verify the therapeutic effect of chemokines that can induce the migration of MSCs on chronic wounds, Yoon et al. developed an IL-8 or macrophage inflammatory protein-3α (MIP-3α)-loaded gelatin-hydroxyphenyl propionic acid (GH) hydrogel. This GH hydrogel achieved the encapsulation of IL-8 and MIP-3α during the *in-situ* polymerization process and maintained the vitality of chemokines. *In vivo* experiments using a 1.0 cm diameter round wound in diabetic mice confirmed that IL-8, which is involved in recruiting a variety of cell types, including MSCs and endothelial cells, had a stronger repair-promoting ability than MIP-3α. Further histological and immunohistochemical results indicated that the restoration of regeneration ability was due to the enhancement of neovascularization (Yoon et al., 2016). Col-based hydrogels have the advantages of high water absorption, good biocompatibility, and low antigenicity. Col also enhances granulation tissue and neovascularization by eliminating high concentrations of MMPs (Simpson et al., 2007; Laghezza Masci et al., 2019). Alginate is an anionic polysaccharide that can form hydrogels under very mild conditions and in the absence of organic solvents (Lee and Mooney, 2012). In addition, alginate effectively prevents dehydration and has long been regarded as an excellent biomaterial for use in wound healing dressings. Alginate hydrogel can be used as a drug delivery carrier to solve the problems of low bioavailability and poor stability of large molecules such as proteins and growth factors (Momoh et al., 2015). For instance, calcium alginate hydrogel has served as a drug carrier for protamine with a neovascularization function in the treatment of chronic wounds (Wang T. et al., 2019). A CS based-dressing was also used to deliver small interfering RNAs (siRNAs) with ECM remodeling function. This RNA interference therapy also strongly promoted vascular regeneration of chronic wounds (Castleberry et al., 2016). For more detail, see section “Extracellular Matrix Remodeling” below.

**Figure 4:**
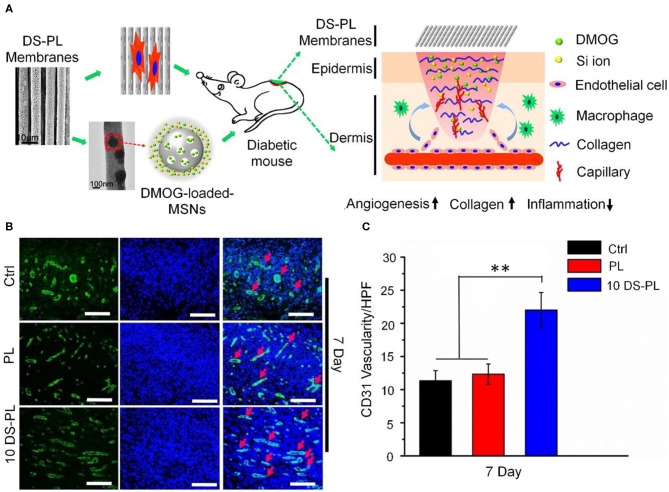
DMOG-loaded PLLA electrospun membrane dressing promotes chronic wound angiogenesis. **(A)** Schematic of material preparation and angiogenesis-promoting mechanism. **(B)** Immunofluorescence CD31 staining images of wounds 7 days post-operation. Scale bar, 100 μm. **(C)** Quantitative analysis of the results shown in **(B)**. **P < 0.01. Reproduced with permission from Li et al. (2018).

In addition to hydrogels, oriented electrospun fibers dressing can also serve as a delivery system for pro-angiogenic drugs for the treatment of chronic wounds. Dimethyl xalylglycine (DMOG) is a small molecule that promotes angiogenesis. To solve the problem of its rapid inactivation in the body, Ren et al. used nano-mesoporous silicon as an intermediate carrier to prepare a DMOG-loaded poly (*L*-lactic acid) (PLLA)-oriented electrospun dressing. As shown in Figure 4, this drug-loading system achieved slow release of DMOG and Si ions; the oriented electrospun fibers regulated the arrangement of cells and the signal transmission mechanism of special phenotype expression through physical cues guided by orientation. *In vitro* tests confirmed that it increased gene expression levels in human umbilical vein endothelial cells, and *in vivo* experiments showed that it promoted wound healing and neovascularization in diabetic mice (Ren et al., 2018).

### Extracellular Matrix Remodeling

The reduction of locally related ECM is an important cause of chronic wound non-healing. Col is the most important component of ECM in skin. Increasing the content and the stability of Col fibers accelerates wound healing. Compared with type III Col, type I Col has better tensile properties. Type III Col is mainly formed in the early stages of wound healing, and the ratio of these two types of Col has a greater impact on the wound healing and the skin quality after healing (Rhett et al., 2008). Mechanistic studies have shown that the reduction in ECM content observed in chronic wounds is related to the overexpression of ECM proteases, such as MMP-9. Decreased ECM results in impaired granulation tissue formation and epithelial formation (Hayden et al., 2011; Castleberry et al., 2016). Therefore, strategies to promote wound healing by optimizing the ECM have gradually attracted attention in recent years.

Caetano et al. confirmed that the polyelectrolyte complex obtained by cross-linking of CS and alginate. The cross-linked polyelectrolyte compound combining the characteristics of CS and alginate reduced the infiltration of neutrophils, promoted the proliferation of fibroblasts, promoted Col formation, and improved the chronic wound microenvironment (Caetano et al., 2015).

An important strategy for ECM remodeling during chronic wound healing is the direct application of Col-based dressings. The components of the dressing can be directly degraded by MMPs, thereby reducing the consumption of Col in the ECM. The 3D structure provided can provide space for vascular regeneration and keratinocyte migration, effectively promoting wound repair (Liden and May, 2013; Bohn et al., 2016).

**Figure 5:**
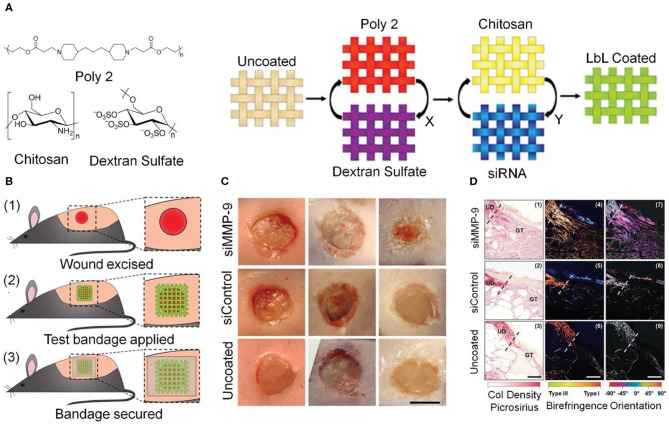
Silencing the MMP-9 gene accelerates the healing of chronic wounds through ECM remodeling. **(A)** Chemical structures of the materials used in the dressing, and schematic diagram of the double coatings fabricated by LbL self-assembly; X and Y represent the number of layers of Poly 2/DS and CS/siRNA, respectively. **(B)** Schematic of the *in vivo* experiments. **(C)** Post-operative photographs of wounds areas. Scale bar, 5 mm. **(D)** Sirius Red staining images of Col tissue infiltration and fusion with wound edges. UD, undamaged dermis. GT, granulation tissue. Scale bars, 75 μm. Reproduced with permission from Castleberry et al. (2016).

Local silencing of sequence-specific MMP genes using RNA interference is another important strategy for regulating MMP, which avoids the disadvantages of using MMP inhibitors such as low efficiency and side effects on skeletal muscle (Overall and Kleifeld, 2006). Castleberry et al. used layer-by-layer (LbL) self-assembly technology to fabricate two coatings on commercial nylon bandages to deliver an MMP-9-specific siRNA (siMMP-9). As shown in Figure 5, the first layer of degradable coating material was poly(β-amino ester) 2 (Poly 2)/dextran sulfate (DS); the second coating was CS with siMMP-9. The drug release properties and silencing of MMP-9 were optimized by adjusting the number of layers of coatings. *In vivo* experiments confirmed that this siMMP-9-loaded (Poly 2/DS)/CS film had a good ability to promote wound healing in diabetic mice and caused a steady increase in Col content. These results demonstrate the therapeutic potential of silencing MMP-9 gene expression in chronic wounds (Castleberry et al., 2016).

### Others

In addition to the above commonly used functional dressings, other dressings, such as those used for diabetic foot neuropathy and scars, also need attention.

Diabetic neuropathy-related ulcers often occur on the plantar surface, and local ulcers often occur on parts of the foot that are under long-term pressure. The main clinical treatment is to use pressure-reducing shoes (Zimny et al., 2003; Clements et al., 2017; Feldman et al., 2017). Zimny et al. used a commercial felted foam dressing to treat patients with neuropathic DFUs. This felted foam dressing contained a thicker foam layer to relieve pressure on the sole of the foot and a thin sticky layer. In their retrospective study, patients using the felted foam dressing achieved the same treatment results as those using traditional clinical therapies (Zimny et al., 2003).

The formation of chronic wound scars often presents a trend of increasing within a year after the wounds healing. Scar formation often leads to repeated ulceration, infection, and even cancer of the wounds. Some researchers have added epidermal growth factor and silver sulfadiazine to silk protein biomaterials to make dressings. The material encapsulates the drug in three forms: a silk membrane, a layered porous silk membrane, and an electrospun nanofiber. It promotes the proliferation of dermal cells and the synthesis of Col, and reduces the formation of scars, thereby providing a feasible strategy for the treatment of chronic wounds (Gil et al., 2013).

## Conclusions and Prospects

In the past two decades, functional biomaterials that can change the wound microenvironment, such as the continuous inflammation, have had a profound impact on the development of dressings for the treatment of chronic wounds. However, many basic studies have been carried out in rodent models, which are characterized by a predominance of M2 macrophages and tend to contracture healing. Thus, the results of these studies need to be further verified using large mammalian models. Current commercial dressings do not meet the needs of patients with chronic wounds, and cost-effective alternative designs containing various types of materials should be developed. Given the clinical problems of patients with chronic wounds, an important research direction is the development of diagnostic functional dressings that clarify the condition of the wound, especially the early symptoms of the subcutaneous tissues with temporary intact skin. As the skin healing process represents the repair process of most tissues, it is widely used in orthopedics and nerve repair technologies, such as 3D printing, and is also suitable for chronic wounds. Therefore, according to the actual characteristics of the patient's wound, such as the position, size, depth, and surrounding tissue of the wound, a more diverse and personalized functional dressing can be produced using computer-aided design. In short, as the application of functionalized biomaterials in chronic wounds is further developed, their therapeutic effects on chronic wounds will be greatly improved, and these materials will replace more clinically invasive operations.

## Author Contributions

XZ wrote the manuscript. XZ, WS, QY, and WQ revised the manuscript. YW and RL designed this work of review and revised the manuscript.

## Conflict of Interest

The authors declare that the research was conducted in the absence of any commercial or financial relationships that could be construed as a potential conflict of interest.
